# Histological Study on the Thickness of Gray Matter at the Summit and Bottom of Folium in Different Age Groups of Bangladeshi People

**DOI:** 10.7759/cureus.42103

**Published:** 2023-07-18

**Authors:** Nahida Sultana Nitu, Seheli Zannat Sultana, Ahsanul Haq, Sharmin A Sumi, Sanjib Kumar Bose, Susmita Sinha, Santosh Kumar, Mainul Haque

**Affiliations:** 1 Anatomy, Khulna City Medical College, Khulna, BGD; 2 Anatomy, Mymensingh Medical College, Mymensingh, BGD; 3 Statistics, Gonoshasthaya-RNA Molecular Diagnostic and Research Center, Dhanmondi, BGD; 4 Anatomy, Bangabandhu Sheikh Mujib Medical University (BSMMU), Dhaka, BGD; 5 Anatomy, Gonoshasthaya Samaj Vittik Medical College, Savar, BGD; 6 Physiology, Khulna City Medical College and Hospital, Khulna, BGD; 7 Periodontology and Implantology, Karnavati School of Dentistry, Karnavati University, Gandhinagar, IND; 8 Karnavati Scientific Research Center (KSRC), School of Dentistry, Karnavati University, Gandhinagar, IND; 9 Pharmacology and Therapeutics, National Defence University of Malaysia, Kuala Lumpur, MYS

**Keywords:** greyish nervous tissue, cerebellum, breadth, examining, staining, sectioning, organs, tissues, research, microscopic

## Abstract

Context

The cerebellum is a part of the hindbrain and consists of cortical gray matter (GM) at the surface and a medullary core of white matter (WM). The GM contains a cell body of neurons that helps process and transmit any command type through nerve fibers found in the WM. The main functions of GM in the central nervous system empower persons to control motor activity, recollection, and passion. So, this research aims to assess the thickness of GM at the summit and bottom of folia by histologically studying the cerebellum cortex.

Methods

The collection of data was a descriptive type of cross-sectional study. The method was the purposive type. This study was conducted from August 2016 to March 2017, and the research was carried out at Mymensingh Medical College's Department of Anatomy, Bangladesh.

Specimens containing cerebellum were preserved from Bangladeshi cadavers according to sexes and ages ranging in years. We chose fresh specimens from people who died within the last 12 hours and preserved them in 10% formol saline. The size of the tissue that was collected for the histological study was not more than 2 cm^2 ^and not more than 4-5 mm thick. Then the tissue was placed in 10% formol saline. This fluid was used for quick fixation and partial dehydration of the tissue. After dehydration, each tissue segment is processed for infiltration and embedding separately. Every section was stained with hematoxylin and eosin stain (H&E) before being coated with dibutyl phthalate polystyrene xylene (DPX) coverslips on slides.

Result

The mean (±SD) thickness of GM at the summit of folium was 886.2±29.7µm in Group A, 925.2±25.9µm in Group B, 912.7±22.3µm in Group C, and 839.9±40.7µm in Group D. Mean (±SD) GM thickness at the bottom of the fissure was 395.6±12.2 µm, 403.9±26.0µm, 380.4±23.4 µm, and 375.8±28.8 µm in Groups A, B, C, and D respectively.

Conclusion

The thickness of the cortex is an essential factor in the normal development process, and it was similar in the current study. Normal aging, Alzheimer's disease, and other dementias cause reduced GM which makes the cortical sheet thin. Huntington's disease, corticobasal degeneration, amyotrophic lateral sclerosis, and schizophrenia are all examples of neurological disorders. Cortical thinning is typically locally localized, and the progression of atrophy can thus disclose much about a disease's history and causal variables. The present study correspondingly found that GM was reduced after the age of 50 years onward. Furthermore, longitudinal investigations of cortical atrophy have the potential to be extremely useful in measuring the efficacy of a wide range of treatments.

## Introduction

The cerebellum is the part of the hindbrain positioned in the cerebellar fossa in the occipital region [1]. The occipital lobe of the cerebrum and cerebellum is separated by a dural fold named the tentorium cerebelli [2]. The cerebellum comprises a cortical gray matter (GM) at the surface, and a medullary core contains white matter (WM) [3,4]. The cerebellar cortex has two distinctive features; it is entirely organized and uniform in structure, and the integral neurons and their processes are arranged in a geometrical configuration [5]. The cerebellum's cortex consists of three layers: external molecular, middle Purkinje, and inside granular [6]. However, GM has the most substantial physiological responsibility and assignment, which permits our day-to-day activity [4,7-9]. The cortex WM and GM are both found in the brain and the spinal cord [10-13]. It is because of the high concentration of neuronal cell bodies in GM; thereby, it looks or is colored grey [4]. The presence of neuronal cell bodies in GM empowers the information development process and releases new data via axons [4,14]. Axons transmit the signal through the WM [4,14]. The GM will enable individuals to control their actions, memories, and emotions [4,15,16]. Numerous brain areas oversee different processes, and GM is crucial in every aspect of human life [4,17]. Variations of GM are associated with physical and cognitive frailty seen in personalities with multiple sclerosis [18-20]. So, if there is any damage to GM, structures can be linked with weakening specific functions [4,21,22].

The cortical thickness has piqued the curiosity of researchers studying normal development and a wide range of neurodegenerative and mental illnesses-variations of the thickness of GM that can manifest in normal aging [23,24]. Cortical thinning is related to the atrophy of brain material, which can reveal much about the evolution and contributory factors of diseases [25], for example, Alzheimer's disease (AD) [26,27] and other dementias [27,28], Parkinson's disease [26,28-30], Huntington's disease [31,32], corticobasal degeneration [33,34], amyotrophic lateral sclerosis [35,36], and schizophrenia [37,38]. Multiple studies revealed that reduced cortical width is a sign of neurodegeneration associated with obesity, high body mass index (BMI), profound psychiatric illnesses, and AD. It has been generally noticed in thinning bilateral frontal and temporal areas, basal nuclei, and cerebellum [39-42].

Multiple systematic reviews and metanalysis reported that the mass of GM reduced has been identified through magnetic resonance imaging (MRI), functional MRI (fMRI), and whole-brain voxel-based morphometry in several brain disorders such as post-traumatic stress disorder, psychosis, bipolar disorder, conduct problems, first-appearance serious depressive diseases, spinocerebellar ataxia type 2, sleep apnea, narcolepsy, escalating Parkinson’s disease, etc. [43-50].

This atrophy GM is often accompanied by a drop in WM volumes and a spike in cerebrospinal fluid gaps [51]. Based on postmortem studies, the modifications to histology underlying these age-related tiny particles variations are more likely due to a loss of neuropil associated with a reduction in dendrites and synapses, along with a loss of nerve-fibers, rather than a direct loss of neurons which becomes relatively limited with age [52-55].

Objectives of the study

The present research aims to estimate the thickness of GM at the summit and bottom of folia by histologically studying the cerebellum cortex. This study may be helpful for physicians and surgeons in treating and evaluating hindbrain disorders. It is also beneficial for radiologists to properly diagnose hindbrain diseases by X-ray, MRI, computerized tomography (CT) scan, and ultrasound scan and helpful for pathologists to diagnose various hindbrain diseases with the knowledge of histological features.

## Materials and methods

**Figure 1:**
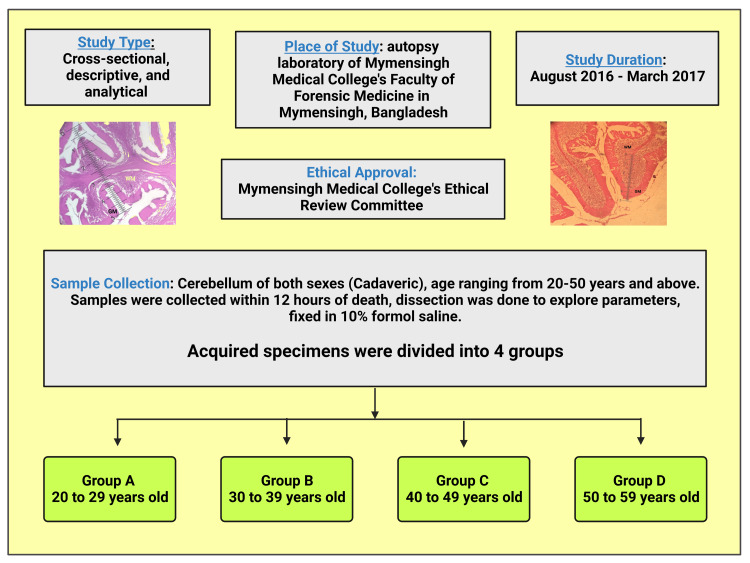
Chart showing the materials and methods of the study. Notes: This figure has been drawn utilizing the premiere version of BioRender with the license number LC25GH1DBA. Image Credit: Susmita Sinha.

This study was cross-sectional, descriptive, and analytical, and the sample collection was purposive. The current study was conducted from August 2016 to March 2017. The cerebellum was taken from Bangladeshi cadavers of both sexes, aged 20 to 50 years and above, at the autopsy laboratory of the Department of Forensic Medicine, Mymensingh Medical College, Mymensingh, Bangladesh. The study obtained ethical approval from the Institutional Review Board (IRB) of Mymensingh Medical College, Bangladesh with Reference No.: MMC/EC/2016/105: Dated: November 30, 2016. The samples for the current study were collected from individuals who passed away within the last 12 hours. These deceased individuals were medico-legal cases. Enough care was taken to conduct the dissection of the cadaver to obtain the necessary parameters (GM), and the latter specimens were fixed in 10% formol saline. The acquired specimens were divided into four groups: A (20 to 29 years old), B (30 to 39 years old), C (40 to 49 years old), and D (50 to 59 years old) (Figure 1).

Processing of tissue

The size of the tissue that was collected for histological study was not more than 2 cm^2^ and not more than 4-5 mm thick. Then the tissue was placed in 10% formol saline, about 15-20 times the tissue volume, for about 18 hours. This fluid was used for quick fixation and partial dehydration of the tissue. Each tissue segment was rinsed in tap water and dehydrated, infiltrated, and embedded separately, following fixation. The blocks were cut into six-micron-thick portions. Each component was stained with hematoxylin and eosin stain (H&E) on slides and coated with coverslips by DPX. As a result, permanent slides for microscopic examination were created.

Measurement of the thickness of GM at the summit and bottom of the fissure

**Figure 2:**
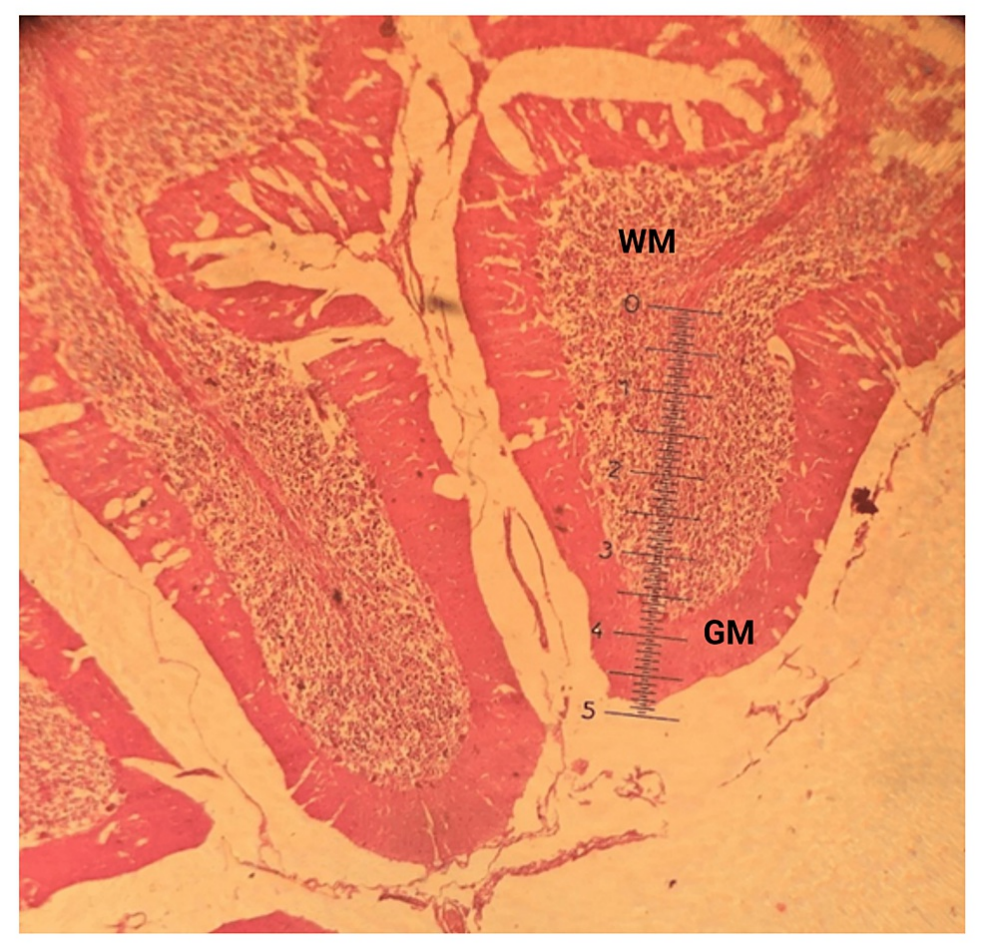
Photomicrograph of the method for evaluating the GM thickness at the folium summit with an ocular micrometer (H & E stain, X 4 objective, and X 10 eyepiece). WM: White matter; GM: gray matter

**Figure 3:**
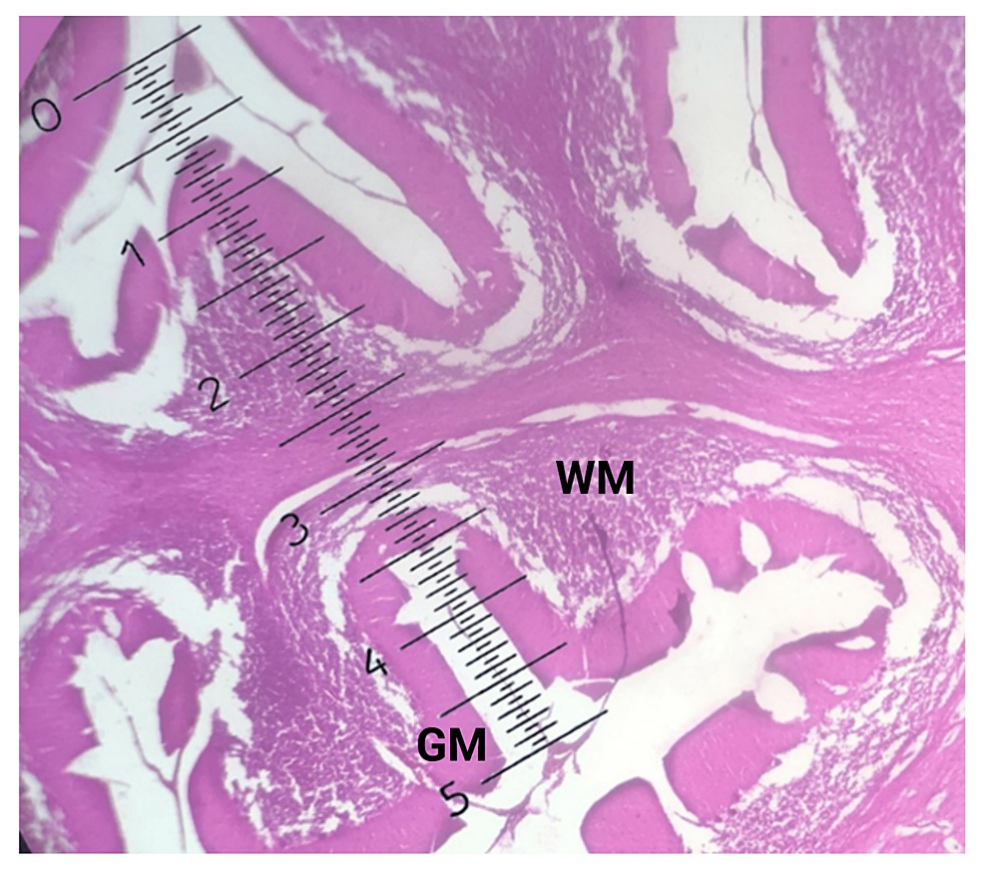
Photomicrograph of the GM width measuring at the bottom of the fissure with an ocular micrometer (H & E stain, X 4 objective, and X 10 eyepiece). WM: White matter; GM: gray matter

Ten slides were taken from each age group from 10 different cerebellums. A total of 40 slides were prepared and examined under a microscope comprises of 10x eyepiece and four objective lenses. A straight line was drawn through the middle of each slide to divide it into two fields for measurement. Two folia were chosen from each field for the highest and lowest GM thickness at the folium's summit and bottom of the fissure. Then the mean value of the density (thickness) of GM at the peak of the folium (Figure 2) and the bottom of the fissure was taken and it was put down in a tabulated form (Figure 3).

Statistical analysis plan

Skewness, kurtosis, and q-q plot were utilized to examine the data distribution and normality. The average distribution and standard deviation of GM thickness at the summit of the folium and bottom of the fissure were presented across stratified age groups. The univariate regression model was used to compare different age groups and densities or thickness. Linear regression models investigated the relationship between gender, height, and GM thickness at the folium's summit and the fissure's bottom. To assess the mean difference in the thickness based on sex, independent sample t-tests were used. A p-value of less than 0.05 was considered statistically significant. The statistical evaluation was performed with STATA-15, Version 15 (Released 2017; StataCorp LLC; College Station, Texas, USA), and graphical representations were created using GraphPad Prism 8.3.2.

## Results

The analysis revealed that the GM thickness at the folium summit exhibited higher values in Group B (925.2±25.9) than in other age groups. However, the thickness began to decline in the age group of 40-49 years (912.7±22.3) and further decreased in Group D (839.9 ± 40.7) (Table 1).

**Table 1:** Distribution of thickness of GM at the summit of folium and bottom of the fissure among the different age groups of studied subjects. Data were presented as mean±SD; GM: gray matter

	Group A (age 20-29 years) (n=10)	Group B (age 30-39 years) (n=10)	Group C (age 40-49 years) (n=10)	Group D (age 50-59 years) (n=10)
The thickness of GM at the summit of folium	886.2±29.7	925.2±25.9	912.7±22.3	839.9±40.7
The thickness of GM at the bottom of the fissure	395.6±12.2	403.9±26.0	380.4±23.4	375.8±28.8

**Figure 4:**
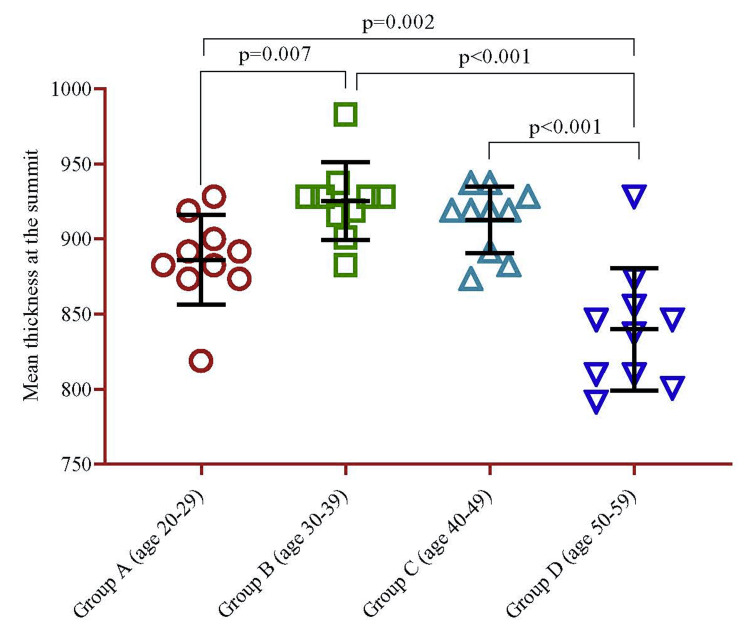
Mean difference of thickness of GM at the summit of folium between the different groups stratified by age. The univariate p-value was calculated using a regression model. GM: Gray matter

Notably, Group D exhibited a significant difference compared to all other groups. Conversely, when compared to Group B, Group A had much less thickness (p=0.007) (Figure 4).

**Figure 5:**
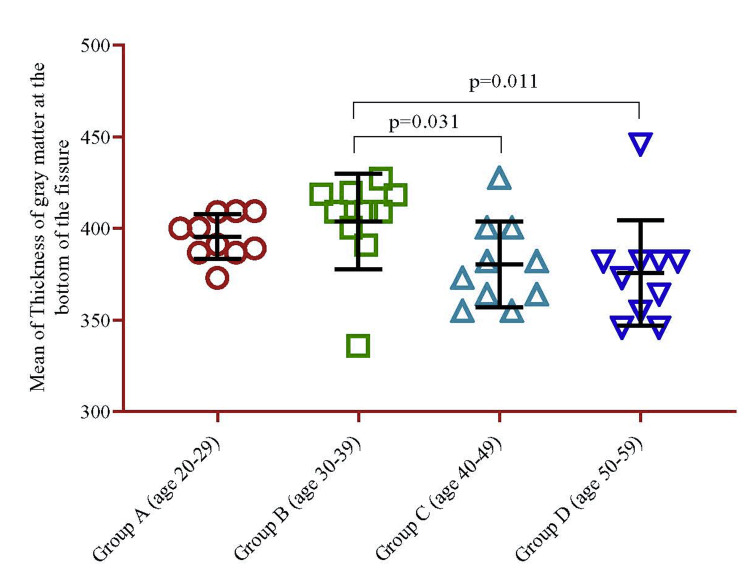
Mean difference of the thickness of GM at the bottom of the fissure between the groups stratified by age. The p-value was calculated using the univariate regression model. GM: Gray matter

The analysis indicated that the GM thickness at the bottom of the fissure was higher in Group B (395.6±12.2), and it began to decline after this age range. The age group of 50-59 years had the lowest thickness (375.828.8) (Group D) (Table 1). Significant mean differences (p=0.011) were between Group D and Group B, as well as between Group B and Group C (p=0.031) (Figure 5).

Table 2 shows the findings of a linear regression analysis that looked at the relationship between GM thickness at different sites (summit and folium of cerebellum) in the brain and two predictor variables: height and gender. The coefficients (β) represent the estimated effect of each predictor variable on the thickness of GM, along with their corresponding 95% confidence intervals (CI). The height variable significantly negatively affected the depth of GM at the top of the folium, with a coefficient of -31.0 (-78.4, -0.19) and a p-value of 0.049. However, the height variable did not approach statistical significance for the layer of GM at the bottom of the fissure, with a coefficient of 23.3 (-3.35, 50.0) and a p-value of 0.065.

**Table 2:** Linear regression association of height and sex with both the thicknesses among the studied subjects. The p-value was calculated using the linear regression model. Bold denotes statistically significant. This explains the findings of a linear regression analysis of the relationship between GM thickness at different sites in the brain and two predictor variables: height and gender. GM: Gray matter; CI: confidence interval

	GM thickness at the summit of folium		GM thickness at the bottom of the fissure	
	β Coff (95% CI)	p-value	β Coff (95% CI)	p-value
Height	-31.0(-78.4, -0.19)	0.049	23.3(-3.35, 50.0)	0.065
Sex				
Female	Ref.		Ref.	
Male	-19.5(-47.6, 8.59)	0.168	12.9(1.34, 28.8)	0.045

Regarding sex as a predictor variable, the analysis used females as the reference category. For the GM thickness at the summit of folium, males showed a coefficient of -19.5 (-47.6, 8.59) with a p-value of 0.168, suggesting a non-significant trend toward thinner GM compared to females. However, for the GM thickness at the bottom fissure, males exhibited a significant positive effect with a coefficient of 12.9 (1.34, 28.8) and a p-value of 0.045, indicating that males tend to have thicker GM than females.

**Figure 6:**
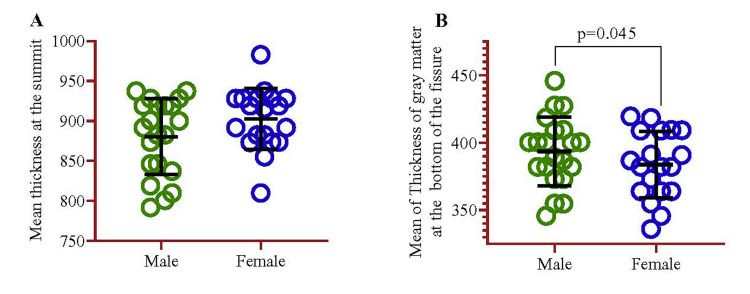
Mean difference of thickness of summit and GM at the bottom of the fissure between sex. The p-value was calculated using an independent sample t-test. GM: Gray matter

In addition, we examined the average disparity between the summit and GM at the base of the fissure based on gender. It was observed that male participants exhibited a significantly greater thickness of GM at the bottom of the fissure (p=0.045) (Figure 6).

## Discussion

The thickness of GM at the summit of folium

The current study revealed that the GM thickness at the folium summit exhibited higher values in the age group of 30-39 years than in other age groups. However, the thickness declined by 40-49 years and decreased at age 50-59. Remarkably, 50-59 (Group D) displayed a statistically significant (p=0.007) difference compared to all other groups. It has been reported that age and sex substantially influence the global brain volume. Additionally, in earlier studies, an extensive decline in the bulk of GM has been noticed in frontal, insular, and cingulate cortices associated with both sexes [56]. Multiple studies revealed that the cerebellar cortex width and sulcus depth are indicators of the progression of several neurological disorders such as Parkinson's disease, AD, multiple sclerosis, and dementia-related issues [57-60]. GM breadth and width reveal that the mean overall volume affects the structure and function of the cerebellum and medulla spinalis in many ways, indicating how the cerebellum is vital in memory, gait control, urine control, and thinking processes [4,61,62]. Neuroanatomy, clinical practice, and neuroimaging research have demonstrated that the cerebellum participates in cognitive tasks [63-65]. The cerebellum governs emotions, executive functions, language, music, working memory, and other neural processes [66,67].

Haque (2010) observed that the mean (±SD) thickness of GM at the foliar summit was 454 (138.85 ± 20) µm in the first group (28-42 weeks of gestation), 924 (224.35 ± 18) µm in the second group (0-30 years) and 905 (117.25 ± 20) µm in the third group (30-60 years) [68]. Yesmin et al. found that the mean thickness of GM and WM at the summit of folium was 510.28 ± 52.98 µm and 240.71 ± 33.31 µm in the age group of 20-29 years, 668.00 ± 36.29 µm and 311.42 ± 16.64 µm in the age group of 30-39 years, 579.16 ± 11.68 µm and 208.33 ± 16.47 µm in the age group of 40-49 years, 427.60 ± 17.35 µm, and 159.00 ± 7.87 µm in above 50 years [69]. The current study's findings were consistent with the equivalent age groups in the previous research [69]. The cortex's overall surface area and thickness were 1692±117 cm2 and 2.46±0.11 mm, respectively. Linear regressions demonstrated age-related reductions (p < 0.001) in the total cortical GM volume (r = - 0.59), total surface area (r = 0.34), and average cortical thickness (r = 0.62) [70].

Although the current study sex is a forecaster variable, the exploration used females as the reference category. Nevertheless, it suggested a statistically non-significant trend toward thinner GM among males compared to females. Cortices are thicker in females than in males in the brain including the cerebellum [71]. This earlier study correlates with the present research [71]. However, another study reported that ﻿sex exhibited an impact on general intelligence and cortical thickness in different regions of the brain. Women display substantial relations in the thickness of GM of overall brain matter; especially of prefrontal and temporal regional cortices, while men demonstrate links principally in temporal-occipital areas [72,73]. Cortical thickening of total brain structure in women has been reported to be consistent with the profile of cognitive differences long seen between the sexes, particularly the females who possess an advantage on language tasks [71-74]. This study reveals confirmation that changes in structure in the cerebellar region could support individual children's developmental advancement toward a specific ability to speak [75-78].

GM thickness at the bottom of the cerebellar fissure

The mean thickness of GM at the bottom of the fissure was higher among the age group 30-39 years than the other three groups in the present study. Haque (2010) found that the mean thickness of GM at a depth of fissure was 185 (36±59.01) µm in the first group (28-42 weeks of gestation), 366 (64±93.85) µm in the second group (0-30 years), and 343 (50±68.90) µm in the third group (30-60 years) [68]. Another study reported that granular, Purkinje, and molecular layers were 250-350, 12, and 250-350 μm broad. 

**Figure 7:**
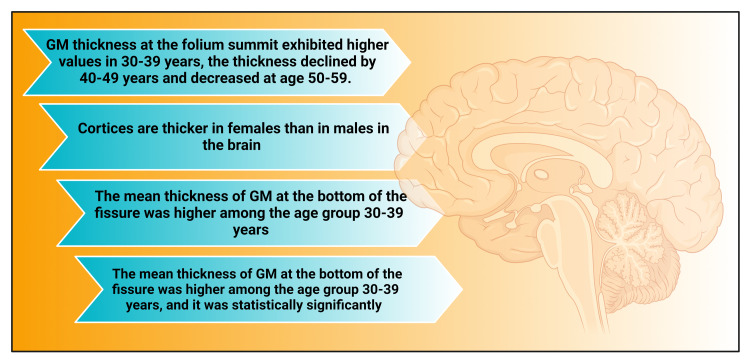
Chart showing the study findings. This figure has been drawn utilizing the premiere version of BioRender with the license number CB25J42B3L. Image Credit: Susmita Sinha. GM: Gray matter

In the current study, males had a statistically significantly higher GM thickness at the bottom cerebellar fissure than females (Figure 7). It has been reported that males had overall larger brains than females. Nevertheless, ﻿territorial sex variances in the GM width and breadth do not remain undetermined throughout the cortical area in a considerable sample size and cover the age assortment from early babyhood to geriatric individuals [79].

Limitations of the study

This study had no financial support. Moreover, the study was conducted on a corpse with no potential reasons for death ascertained earlier. Thereby, higher sophisticated methods to assess cannot be accessed.

## Conclusions

GM exact global measurement throughout the brain was not possible because of several technological, pathological, and physiological reasons. Consequently, it is enlightening to investigate the function of GM shrinkage in the illness process and the impact of interventions to reduce neurodegeneration. The current study's findings and conclusions were expected to provide insight into the thickness of GM and how it varies with age in the Bangladeshi population. These observations may also contribute to standardizing measurements collected by other observers in our country.
